# Feasibility, safety, and efficacy of early prophylactic donor lymphocyte infusion after T cell-depleted allogeneic stem cell transplantation in acute leukemia patients

**DOI:** 10.1007/s00277-023-05145-1

**Published:** 2023-03-07

**Authors:** Boris van der Zouwen, E. A. S. Koster, P. A. von dem Borne, L. E. M. Oosten, M. W. I. Roza-Scholten, T. J. F. Snijders, D. van Lammeren, P. van Balen, W. A. F. Marijt, H. Veelken, J. H. F. Falkenburg, L. C. de Wreede, C. J. M. Halkes

**Affiliations:** 1 Department of Hematology, Leiden University Medical Center, C2R, 2300 RC, Leiden, 9600 The Netherlands; 2 Department of Biomedical Data Sciences, Leiden University Medical Center, Leiden, The Netherlands; 3 Department of Hematology, Medical Spectrum Twente, Enschede, The Netherlands; 4 Department of Hematology, HagaZiekenhuis, The Hague, The Netherlands

**Keywords:** Prophylactic donor lymphocyte infusion, Allogeneic stem cell transplantation, Graft-versus-host-disease, Treatment success

## Abstract

Prophylactic donor lymphocyte infusion (DLI) starting at 6 months after T cell-depleted allogeneic stem cell transplantation (TCD-alloSCT) can introduce a graft-versus-leukemia (GvL) effects with low risk of severe graft-versus-host-disease (GvHD). We established a policy to apply low-dose early DLI at 3 months after alloSCT to prevent early relapse. This study analyzes this strategy retrospectively. Of 220 consecutive acute leukemia patients undergoing TCD-alloSCT, 83 were prospectively classified to have a high relapse risk and 43 were scheduled for early DLI. 95% of these patients received freshly harvested DLI within 2 weeks of the planned date. In patients transplanted with reduced intensity conditioning and an unrelated donor, we found an increased cumulative incidence of GvHD between 3 and 6 months after TCD-alloSCT for patients receiving DLI at 3 months compared to patients who did not receive this DLI (0.42 (95%Confidence Interval (95% CI): 0.14–0.70) vs 0). Treatment success was defined as being alive without relapse or need for systemic immunosuppressive GvHD treatment. The five-year treatment success in patients with acute lymphatic leukemia was comparable between high- and non-high-risk disease (0.55 (95% CI: 0.42–0.74) and 0.59 (95% CI: 0.42–0.84)). It remained lower in high-risk acute myeloid leukemia (AML) (0.29 (95% CI: 0.18–0.46)) than in non-high-risk AML (0.47 (95% CI: 0.42–0.84)) due to an increased relapse rate despite early DLI.

**Supplementary Information:**

The online version contains supplementary material available at 10.1007/s00277-023-05145-1.

## Introduction

Allogeneic stem cell transplantation (alloSCT) is a curative treatment option for acute leukemia patients by donor-derived T cell responses against recipient hematopoietic cells including the malignant cells, also known as the graft-versus-leukemia (GvL) effect [1–4]. GvL is frequently associated with graft-versus-host disease (GvHD), i.e., donor T cell responses against nonhematopoietic recipient tissues. GvHD requiring systemic immunosuppression (sIS) carries significant morbidity and mortality [5]. Of all patients receiving alloSCT for acute leukemia, 30% to 70% require treatment for chronic GHVD, often for longer than 2 years [6–9].

GvHD risk can significantly be reduced by depletion of donor T cells, but T cell depletion (TCD) is associated with an increased relapse rate, especially in high-risk leukemia patients [10–13]. Donor lymphocyte infusion (DLI) after TCD-alloSCT is applied to achieve a persistent GvL response without induction of severe GvHD [12, 14, 15]. The rationale to postpone this DLI is to wait for a less proinflammatory environment than present at the time of the transplantation, which gradually occurs after definitive donor hematopoiesis has been established, tissue damage has been repaired, and recipient antigen-presenting cells (APC) have been partially replaced by donor APC [16]. Other factors that can influence the magnitude of the donor-derived immune response after DLI include the number of infused effector cells and the degree of genetic disparity between patient and donor [17].

Our center previously reported that most patients with acute leukemia experience persistent remission without the need of sIS for chronic GvHD after receiving prophylactic DLI at 6 months after TCD-alloSCT [18]. However, relapses before this DLI occurred in patients with high-risk acute leukemia [19]. Therefore, we adjusted our treatment algorithm in 2007 by adding an extra prophylactic low-dose DLI at 3 months after TCD-alloSCT for this patient group [20]. The aim of this early DLI was to lower the risk of recurrence of leukemia prior to standard prophylactic DLI at 6 months without inducing a significant increase in the risk of severe GvHD.

In this study, we investigated the feasibility, toxicity and long-term efficacy of a strategy in which an early low-dose DLI was scheduled after TCD-alloSCT for all acute leukemia patients with a high early relapse risk and no previous GvHD.

## Materials and methods

### Study population and prophylactic DLI strategy

All consecutive patients who underwent TCD-alloSCT with a 9/10 or 10/10 HLA-matched donor for acute leukemia in complete remission (CR) at the Leiden University Medical Center (LUMC) between January 2007 and December 2015 were included in this study. All patients gave written informed consent for treatment, data collection, and scientific evaluation before transplantation. The study was approved by the LUMC Ethics Committee. Data were analyzed as of February 2021. Patients who were transplanted for AML after a myeloproliferative disease or were planned to receive experimental cell products after transplantation as part of a clinical trial were excluded from this analysis.

Since 2007, all patients with acute leukemia were scheduled to receive prophylactic DLI, defined as an infusion that is planned to be administered at a prescheduled time point after TCD-alloSCT to patients without a hematological relapse, independently of chimerism status [21]*.* All patients were planned to receive 1.5 or 3 × 10^6^ CD3 cells/kg, for unrelated and matched sibling patient-donor combinations, respectively at 6 months. Patients who developed GvHD before this timepoint, did not receive DLI as the occurrence of GvHD was interpreted as indication of an alloimmune response. High-risk leukemia patients were scheduled to receive a low-dose prophylactic DLI at 3 months after transplantation as well (0.15- or 0.3 × 10^6^ CD3 cells/kg, for unrelated and matched sibling patient-donor combinations, respectively) [20]. Since DLI is standard care in this strategy, donors are informed that a request for T cell apheresis would probably follow some months after the donation of the stem cells. T cell apheresis for multiple DLI products was performed immediately prior to the first DLI. Fresh donor T cells were administered as the 1^st^ DLI, and remaining T cells were cryopreserved for subsequent DLI in escalating doses. To compensate for cell loss during the freezing and thawing procedure, a double dose of T cells was frozen for every subsequent infusion. Prophylactic DLI was withheld or canceled in the presence of relapse, active GvHD, concomitant severe infections, or inflammatory diseases necessitating hospital admission.

High risk of early relapse with respect to our DLI strategy was defined according to applicable national Dutch recommendations for acute myeloid and lymphoblastic leukemia [20–23]. Specifically, high-risk ALL was defined by high leukocyte count at diagnosis (> 30 × 10^9^/L in B-ALL and > 100 × 10^9^/L in T-ALL), failure to achieve CR after the first induction therapy, and/or unfavorable karyotypes (*t*(9;22), *t*(4;11), hypodiploidy, or complex abnormalities). High-risk AML was defined by therapy-related AML, presence of monosomal karyotype and/or abn3q26 (EVI1), persistence of genetic abnormalities despite morphologic CR at time of alloSCT, and/or relapsed acute leukemia after previous curative induction chemotherapy. Leukemia patients not fulfilling these high-risk criteria served as the control group for this analysis.

### Study endpoints

See supplement II for detailed descriptions of all study endpoints.

The primary endpoint for the feasibility analysis was defined as the percentage of patients with high-risk leukemia who received the requested first prophylactic DLI at 3 or 6 months after transplantation. Primary outcome for the toxicity analysis was the cumulative incidence of moderate to severe GvHD in the period between the first and second prophylactic DLI. To evaluate whether this toxicity was due to the low-dose DLI, the intervention group was compared to a control group, consisting of patients being alive and without relapse or GvHD at 3.25 months who were not intended to receive the low-dose DLI because they lacked the criteria for high-risk acute leukemia. As the median time between the first and second DLI was 3.12 months for the intervention group, for this analysis, the follow-up time for the patients who did not receive the second prophylactic DLI was stopped 3.12 months after the first DLI to keep the at-risk periods equal. Treatment success was defined as being alive without previous relapse post-alloSCT or current use of sIS.

### Statistical analysis

Time was measured from the transplantation date, DLI (intervention group), or the 3-month index date (3.25 months, control group). RFS was defined as time from transplantation to relapse or death, whatever occurred first, with patients censored at the last follow-up visit if they were relapse-free. Probabilities of OS and RFS with associated 95% confidence intervals (95% CI) were calculated by the Kaplan–Meier method. Median follow-up was estimated by the reverse Kaplan–Meier method. Cumulative incidences of relapse and NRM were estimated together in one competing risks model. The cumulative incidence of GvHD was estimated in a competing risks model with relapse, DLI, and death as competing risks. The probability of treatment success at 1, 3, and 5 years after alloSCT was calculated using a Markov multistate model. See supplemental III for detailed information.

## Results

### *Collection offreshly harvested donor lymphocytes for prophylactic infusions starting at 3 months after alloSCT is feasible*

Of the 220 acute leukemia patients, 83 (Table 1) fulfilled the criteria for high-risk leukemia at time of transplantation and were scheduled to receive an early DLI. All patients engrafted with a median time to neutrophil recovery of 15 days (range 9–48 days). Of these 83 patients, 43 were in continuous CR and eligible for DLI at 3 months after alloSCT. From 1 sibling donor, T cells had already been harvested and cryopreserved before transplantation due to expected unavailability of this donor after transplantation. Therefore, DLI was requested for 42 patients. For 41 patients, DLI was obtained within a median of 3 weeks after the requesting date. One unrelated donor was unavailable.

**Table 1:** Baseline characteristics of the high-risk group (83 patients)

Variable	Categories	Frequency	Percentage
Sex	Male	56	67%
	Female	27	33%
Type of leukemia	AML	45	54%
	ALL	38	46%
Age at alloSCT	Median (years)	50	(range: 18–72)
	< 45 yr	34	41%
	45-55 yr	23	28%
	55-65 yr	16	19%
	> 65 yr	10	12%
Indication high-risk	ALL: high WBC at diagnosis	13	16%
	ALL: no CR1	7	8%
	ALL: unfavorable karyotype	18	22%
	AML: Th-AML	13	16%
	AML: monosomal karyotype	7	8%
	AML: EV1/abn3q26	14	17%
	AML: no cytogenetic CR	4	5%
	AML: progressive disease during remission-induction cycles	7	8%
Donor relation	HLA-identical sibling	29	35%
	Unrelated	54	65%
10 out 10 matching	Yes	70	84%
	No	13	16%
Conditioning regimen	MA	58	70%
	RIC	25	30%
Time diagnosis to alloSCT	Median (months)	6.2	(range: 3.3–55.1)
Stem cell source	PBSCT	79	95%
	BMT	4	5%
Stem cell quantity	Median (CD34 × 10^6^/kg)	7.2	(range: 1.4–28.3)
T cell depletion in vitro	Campath in the bag	81	98%
	CD34 selection	2	2%
T cell depletion in vivo	None	22	27%
	Campath	44	53%
	ATG + Campath	17	20%
GvHD prophylaxis after alloSCT	None	46	55%
	Cyclosporine A	37	45%

*WBC* white blood cell count, *CR1* complete remission after first remission-induction cycle, *alloSCT* allogeneic stem cell transplantation, *Th-AML* therapy-related AML, *MA* myeloablative, *RIC* reduced intensity, *PBSCT* peripheral blood stem cells harvested by plasmapheresis after mobilization by granulocyte colony stimulating factor (GCSF), *BMT* bone marrow derived stem cells, *ATG a*nti-T lymphocyte globulin, *GvHD* graft-versus-host-disease

Early DLI was administered to 42 patients (median period after alloSCT 3.25 (range 2.92–4.66) months). In 39 of the 41 patients (95%) for whom fresh DLI was obtained, this was done within 2 weeks of the intended infusion date. The administration of DLI was postponed in 2 patients because of suspicion of developing GvHD (*n* = 1) or because of a deteriorating performance state for which the patient was admitted to the hospital (*n* = 1). For 7 patients of the high-risk group who did not receive DLI at three months, standard DLI was requested, received, and actually administered at six months after alloSCT. In conclusion, these data show that 98% of the requested DLIs were available for scheduled DLI administration starting at 3 months after alloSCT.

### Relapsing disease and nonrelapse mortality interfere with the early DLI strategy

**Fig. 1:**
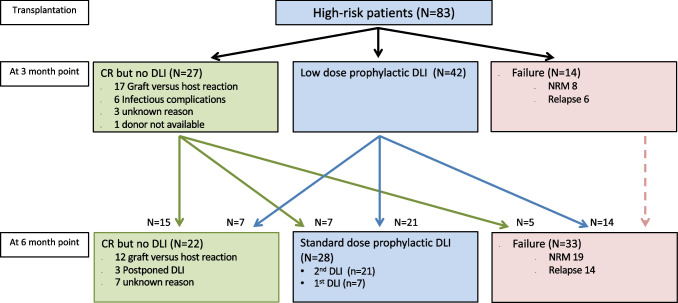
Schematic overview of events in the first 6 months after transplantation for the very poor risk acute leukemia cohort. The 3-month point was defined as the date of administration of the planned low-dose donor lymphocyte infusion (DLI) (median 3.25 months after transplantation, range: 2.92–4.66) for the patients who received this DLI (blue box) and at 3.25 months after transplantation for the patients who did not receive DLI either due to failure due to nonrelapse mortality (NRM) or relapse (red box) or due to other reasons while still in complete remission (CR) (green box). Follow-up of these patients is included until the 6-month point. This period was defined by either the time from transplantation until the date of administration of the planned 6 month DLI (median: 3.12 months after the 3-month DLI) after transplantation (blue box) or at 6.37 (3.25+3.12) months after transplantation for the patients who did not receive DLI either due to failure (red box) or due to other reasons while still in CR (green box)

For 40 of the 83 patients with high-risk leukemia (48%), no low-dose prophylactic DLI was requested (Fig. 1). Six patients had a relapse (3 after Reduced Intensity Conditioning, RIC), and 8 patients (1 after RIC) had died before 3.25 months after alloSCT without relapse. For six patients, no early DLI was requested because they were admitted to the hospital for the treatment of severe infectious complications. In accordance with the strategy, for 17 patients (20%) DLI was not considered to be necessary because of the presence of GvHD after alloSCT for which sIS was given (*n* = 6) or because of signs of active GvH reaction for which only local treatment was necessary (*n* = 11). For the remaining three patients, no specific reasons could be identified for not scheduling early DLI. Of the 42 patients receiving prophylactic low-dose DLI at 3 months, 7 (17%) suffered a relapse and 7 (17%) died without relapse before 6 months. 50% of these 42 patients received a second DLI at 6 months, while 17% did not receive this DLI because of GvHD after the first DLI. Of the 17 patients who did not receive a low-dose DLI at 3 months because of the presence of GvHD after alloSCT, 1 (6%) developed a relapse and 2 (12%) died without relapse between 3 and 6 months.

### High-risk acute leukemia patients transplanted from an unrelated donor after RIC experience additional toxicity after early DLI

**Fig. 2:**
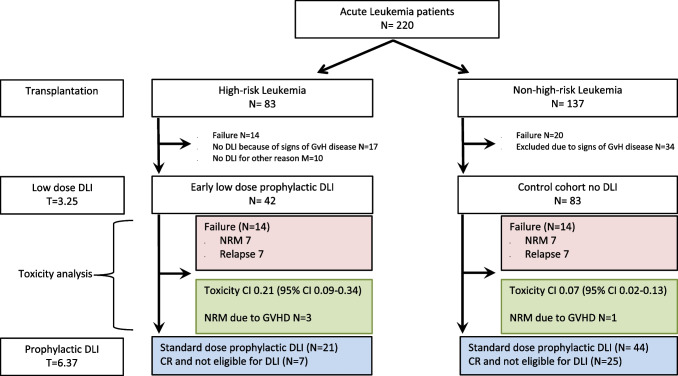
Schematic overview of the intervention and control group selection and of the time period in which the additional toxicity of the low-dose prophylactic DLI administered to the very poor risk acute leukemia patients is evaluated. The groups were defined as described in “Materials and methods.” Follow-up started at the 3-month point (either the date of low-dose DLI administration (intervention group) or at 3.25 months for the patients who did not receive the low-dose DLI (control group)). Toxicity analysis was stopped at failure (nonrelapse mortality (NRM) or relapse, depicted in the red boxes for each group), at administration of the normal-dose prophylactic DLI or at the 6-month point (6.37 months after transplantation, i.e., median time for standard-dose DLI). The cumulative incidences of moderate and severe GvHD between the 3- and 6-month points are given for each group in the green boxes. In these green boxes, the number of patients who died of graft-versus-host-related nonrelapse mortality (NRM) within this toxicity period is shown

To investigate the safety of the strategy of early DLI at 3 months, we examined the additional toxicity due to GvHD developing after this DLI as compared to the toxicity observed in patients in the same time period after alloSCT who did not receive early DLI as they had non-high-risk acute leukemia (83 patients, Fig. 2). Baseline characteristics for both groups are given in Table 2. The cumulative incidence of moderate to severe GvHD between 3 and 6 months was higher in the intervention group compared to the control group, 0.21 (95% CI: 0.09–0.34) versus 0.07 (95% CI: 0.02–0.13). Three patients (7%) in the intervention group died due to GvHD toxicity or infectious complications during the treatment of GvHD after the early DLI, compared to 1 patient in the control group (1%). To examine whether the type of conditioning regimen (RIC vs myeloablative) and genetic disparity (unrelated donor vs 10/10 HLA matched sibling donor) influenced this toxicity, we analyzed these subgroups separately (Table 3). Similar cumulative incidences of GvHD were seen for the intervention and control groups after MA conditioning independent of donor-patient matching. However, after alloSCT with RIC and an unrelated donor, the cumulative incidence of GvHD was significantly higher in the intervention group (0.42 (95% CI: 0.14–0.70); *n* = 12) compared to the control group (0; *n* = 29). All 3 patients who died due to severe GvHD after the early DLI had been transplanted with an unrelated donor, 2 of the 3 after RIC. In conclusion, these data illustrate that additional toxicity due to GvHD can be seen after administration of early DLI in patients receiving grafts of unrelated donors after a RIC regimen.

**Table 2:** Baseline characteristics of the intervention and control cohorts

Variable	Categories	Intervention (*N* = 42)	Control (*N* = 83)	*P* value*
Sex	Male	25 (60%)	39 (47%)	0.185
	Female	17 (40%)	44 (53%)	
Type of leukemia	AML	27 (64%)	72 (87%)	0.003
	ALL	15 (36%)	11 (13%)	
Age at alloSCT	Median (years)	50 (18–71)	54 (20–72)	0.409
	< 45 yr	15 (36%)	29 (35%)	
	45–55Yr	11 (26%)	15 (18%)	
	55–65 yr	10 (24%)	26 (31%)	
	> 65 yr	6 (14%)	13 (16%)	
Donor relation	HLA-identical sibling	17 (41%)	38 (46%)	0.572
	Unrelated	25 (59%)	45 (54%)	
10 out 10 matching	Yes	36 (86%)	74 (89%)	0.576
	No	6 (14%)	9 (11%)	
Conditioning regimen	MA	26 (62%)	38 (46%)	0.089
	RIC	16 (38%)	45 (54%)	
Performance status at alloSCT	WHO performance score 0–1	35 (92%)	70 (90%)	0.684
	WHO performance score ≥ 2	3 (8%)	8 (10%)	
	Missing	4	5	
GvHD prophylaxis	None	29 (69%)	67 (81%)	0.144
	Cyclosporine A	13 (31%)	16 (19%)	

*MA* myeloablative, *RIC* reduced intensity, *alloSCT* allogeneic stem cell transplantation, *GvHD* graft-versus-host-disease. *P values were calculated using for the categorical variables chi-square test and for the continuous values the unpaired *T*-test

**Table 3:** Cumulative incidence of GvHD in subgroup analysis

		Intervention group		Control group
	*N*	Cumulative incidence of GvHD (95% CI)	*N*	Cumulative incidence of GvHD (95% CI)
MA conditioning regimen				
Related	13	0.15 (0–0.35)	22	0.09 (0–0.21)
Unrelated	13	0.15 (0–0.35)	16	0.19 (0–0.38)
RIC conditioning regimen				
Related	4	0	16	0.06 (0–0.18)
Unrelated	12	0.42 (0.14–0.70)	29	0

*MA* myeloablative, *RIC* reduced intensity, *CI* confidence interval, *N* number of patients, *GvHD* moderate to severe graft-versus-host disease

Competing risk analysis for 4 donor-conditioning subgroups. Cumulative incidences of GvHD from DLI/3.25 months until second DLI/6.37 months after transplantation are shown. Death and relapse were taken as competing events

### Long-term outcome of early prophylactic low-dose DLI at 3 months after TCD-alloSCT for high-risk acute leukemia patients

The goal of the strategy with early DLI at three months after TCD-alloSCT in patients with high-risk AML or ALL is to increase the probability of long-term treatment success, defined as being alive without previous relapse post-alloSCT or current use of sIS.

**Fig. 3:**
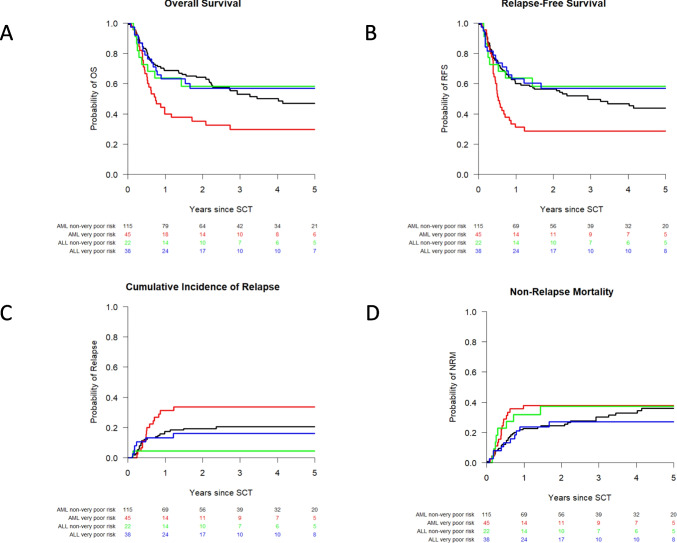
Overall survival, relapse-free survival, cumulative incidences of relapse, and nonrelapse mortality for the whole acute leukemia cohort. Kaplan-Meier curves showing probabilities of **a** overall survival and **b** relapse-free survival and cumulative incidence curves of **c** relapse and **d** nonrelapse mortality based on a cohort of 94 patients with non-very poor-risk AML (black line), 45 patients with very poor-risk AML (red line), 22 patients with non-very risk ALL (green line), and 38 patients with very poor-risk ALL (blue line)

Baseline characteristics of the different cohorts are presented in Supplemental Material Table 1a/b. Median follow-up of patients was 100 months (range: 50–169 months). Only 3 patients were lost to follow-up. Figure 3a–d shows Kaplan–Meier curves of OS and RFS and cumulative incidence curves of relapse and NRM for the different subgroups. Probabilities of OS, RFS, relapse, NRM, moderate to severe GvHD, and treatment success at 1, 3, and 5 years are given in Table 4.

**Table 4:** Probabilities of OS, RFS, relapse, NRM, moderate to severe GvHD, and treatment success at 1, 3, and 5 year after alloSCT

Group (total: *n* = 220)	Categories	Probability 1 yr (95% CI)	Probability 3 yr (95% CI)	Probability 5 yr (95% CI)
AML non-high-risk (*N* = 115)	OS	0.69 (0.60–0.77)	0.55 (0.46–0.64)	0.50 (0.41–0.60)
	RFS	0.61 (0.52–0.70)	0.51 (0.42–0.60)	0.48 (0.39–0,57)
	Relapse	0.17 (0.10–0.23)	0.19 (0.12–0.26)	0.19 (0.12–0.26)
	NRM	0.23 (0.15–0.30)	0.30 (0.21–0.38)	0.33 (0.24–0.42)
	GvHD	0.39 (0.30–0.48)	0.45 (0.36–0.54)	0.45 (0.36–0.54)
	TS	0.50 (0.42–0.60)	0.49 (0.40–0.59)	0.47 (0.39–0.57)
AML high-risk (*N* = 45)	OS	0.40 (0.26–0.54)	0.31 (0.18–0.45)	0.31 (0.18–0.45)
	RFS	0.31 (0.18–0.45)	0.29 (0.16–0.42)	0.29 (0.16–0.42)
	Relapse	0.31 (0.18–0.45)	0.33 (0.20–0.47)	0.33 (0.20–0.47)
	NRM	0.38 (0.24–0.52)	0.38 (0.24–0.52)	0.38 (0.24–0.52)
	GvHD	0.40 (0.26–0.54)	0.40 (0.26–0.54)	0.40 (0.26–0.54)
	TS	0.27 (0.16–0.43)	0.29 (0.18–0.46)	0.29 (0.18–0.46)
ALL non-high-risk (*N* = 22)	OS	0.64 (0.44–0.84)	0.59 (0.39–0.80)	0.59 (0.39–0.80)
	RFS	0.64 (0.44–0.84)	0.59 (0.39–0.80)	0.59 (0.39–0.80)
	Relapse	0.05 (0–0.13)	0.05 (0–0.13)	0.05 (0–0.13)
	NRM	0.32 (0.12–0.51)	0.36 (0.16–0.56)	0.36 (0.16–0.56)
	GvHD	0.41 (0.20–0.61)	0.41 (0.20–0.61)	0.41 (0.20–0.61)
	TS	0.50 (0.33–0.76)	0.59 (0.42–0.84)	0.59 (0.42–0.84)
ALL high-risk (*N* = 38)	OS	0.63 (0.48–0.78)	0.58 (0.42–0.74)	0.55 (0.39–0.71)
	RFS	0.63 (0.48–0.78)	0.58 (0.42–0.74)	0.55 (0.39–0.71)
	Relapse	0.13 (0.02–0.24)	0.16 (0.04–0.27)	0.16 (0.04–0.27)
	NRM	0.24 (0.10–0.37)	0.26 (0.12–0.40)	0.29 (0.15–0.43)
	GvHD	0.24 (0.10–0.37)	0.26 (0.12–0.40)	0.26 (0.12–0.40)
	TS	0.58 (0.44–0.76)	0.58 (0.44–0.76)	0.55 (0.42–0.74)

*alloSCT* allogeneic stem cell transplantation, *CI* confidence interval, *OS* overall survival, *RFS* relapse-free survival, *NRM* nonrelapse mortality, *GvHD* moderate to severe graft-versus-host disease, *TS* treatment success (being alive without relapse or sIS)

The probability of treatment success for the total cohort was 0.47 (95% CI: 0.41–0.54) at 1 year and 0.46 (95% CI: 0.40–0.53) at 5 years after alloSCT. At 1 year 16%, at 3 years 3%, and at 5 years only 1% of the patients who were still alive and in CR needed sIS (Table 2 Supplemental Material). In the subgroup analysis, treatment success at 1 year was 0.50 (95% CI: 0.42–0.60) for standard risk AML patients, 0.27 (95% CI: 0.16–0.43) for high-risk AML, 0.50 (95% CI: 0.33–0.76) for non-high-risk ALL, and 0.58 (95% CI: 0.44–0.76) for high-risk ALL. No major changes in the probability of treatment success took place between 1 and 5 years (see Table 4).

In conclusion, the probability of treatment success in patients with high-risk ALL who have been treated by the strategy of early DLI is similar to that in non-high-risk ALL patients. In contrast, the probability of treatment success in patients with high-risk AML was lower compared to non-high-risk AML patients, especially due to a high relapse probability in the first year.

## Discussion

This study illustrates that it is feasible to commit donors to be available for leukapheresis shortly after transplantation by informing them before the donation of stem cells. Administration of early DLI resulted in an increased cumulative incidence of moderate to severe GvHD between 3 and 6 months, but only in patients transplanted after reduced intensity conditioning (RIC) using an unrelated donor. Since all high-risk leukemia patients in our center were treated by this early DLI strategy, the added value of this strategy in this particular group cannot be quantified, but similar RFS and treatment success at 5 years in high- and non-high-risk ALL patients suggests a beneficial effect. The use of tyrosine kinase inhibitors in the standard treatment schedule for patients with Philadelphia-positive high-risk ALL after transplantation could attribute to the beneficial outcome in this group as well. In high-risk AML patients, however, 5 years treatment success was lower with 0.29 (95% CI: 0.18–0.46) compared to 0.47 in the non-high-risk AML (95% CI: 0.39–0.57). Apparently, the increased incidence of GVHD, leading to an increased NRM did not lead to a sufficiently strong reduction of relapses in this group with high-risk AML.

Strategies to reduce acute GvHD after alloSCT by eradicating or suppressing donor-derived alloreactive T cells will lead to an increased relapse risk [10–12, 22]. Timely infusion of prophylactic DLI after alloSCT can be used to decrease this risk [19]. A prerequisite of this strategy using prophylactic DLI early after alloSCT is that the donor is available to donate fresh lymphocytes soon after donating the stem cells. To circumvent this potential limitation, some centers harvest and cryopreserve the donor lymphocytes at the same time as the stem cells [23, 24]. However, G-CSF administration, which is used to collect peripheral stem cells at that time, influences the composition and the effectiveness of the DLI. The cellular product will contain more myeloid precursor cells which could directly affect the immunologic effects of the donor lymphocytes [23] or the viability of the donor lymphocytes after thawing [25, 26]. Therefore, we preferentially harvest the donor lymphocytes when the first DLI is requested, and virtually all donors were available to donate additional lymphocytes at time.

The risk to develop GvHD after DLI is supposedly higher early after transplantation [27–29]. Therefore, the dose of infused donor lymphocytes at 3 months has been determined to be ten times as low as the dose we administer at 6 months [29]. We observed additional toxicity due to GvHD after early low-dose DLI at 3 months after alloSCT. Subgroup analysis suggests that this was mainly seen in patients who were transplanted after RIC with an unrelated donor compared to their control group, but this observation is based on a limited number of patients We argue this is not due to the genetic disparity since we find a comparable cumulative incidence of GvHD after early low-dose DLI between patients receiving myeloablative conditioning, comparing both donor types. Indicating that the 50% reduction of T cell dose for patients with an unrelated donor is sufficient to counterbalance the increased GvHD risk due to the genetic disparity in this setting. A possible explanation of the increased GvHD risk in this group can be the persistence of recipient antigen presenting cells (APC) in these patients due to the reduced myelotoxicity leading to a mixed chimerism status [24]. This is in line with older experimental data showing that the interaction of donor T-lymphocytes with recipient APC is crucial for the development of acute GvHD [30, 31].

Overall and relapse free survival in our cohort were in line with published data from real life outcomes after alloSCT [32–38]. It is difficult to extrapolate the results to current cohorts as risk classifications have changed over the last years. Nevertheless, this study demonstrates that early low-dose DLI can be administered for additional disease control without introducing GvHD needing long-term sIS treatment. After 1 and 3 years, only 16% and 3% of the surviving patients without relapse still used sIS. This is considerably lower compared to data published of non-TCD-alloSCT [6–8, 39].

To properly assess the burden of GvHD and its treatment, we advocate the use of more dynamic endpoints, which express that patients can go through several episodes of failure and success, besides endpoints like RFS or GvHD-free Relapse-free survival (GRFS), where the patient cannot experience a success after the first failure. Both our group and other groups have developed closely related new outcome measures like current and dynamic GRFS that do more justice to the complex disease-recovery process than traditional outcome measures since they acknowledge that GvHD can be a transient state [8, 18, 40–42]. Large-scale studies incorporating these endpoints are still rare since they require both high quality follow-up data and sophisticated statistical analyses.

In our cohort, no major changes in the probability of treatment success took place between 1 and 5 years. In order to improve the outcome of the strategy of TCD alloSCT followed by prophylactic DLI, NRM and relapse risk in the first year should therefore be decreased. Decreasing the NRM between alloSCT and DLI could be done by the use of less toxic conditioning regimens or by applying different forms of TCD. To avoid excess GVHD-associated mortality after DLI, the dose of T cells in the infusion could be adapted in selected patient groups. Decreasing relapse risk between alloSCT and DLI could be done by additional posttransplant treatment such as hypomethylating agents and venetoclax [43, 44].

In conclusion, we demonstrate that the strategy of T cell-depleted alloSCT followed by low-dose prophylactic DLI at 3 months and standard dose DLI at 6 months is feasible for high-risk leukemia patients, whereas the additional toxicity, due to moderate to severe GvHD, was limited to patients who were transplanted with an unrelated donor after a RIC regimen. Treatment success was comparable in high-risk ALL and non-high-risk ALL, but for high-risk AML patients, treatment success remained inferior compared to the other groups.

## Supplementary Information

Below is the link to the electronic supplementary material.

Supplementary file1 (DOCX 18 kb)
