# Supplementary material for: Feasibility, safety, and efficacy of early prophylactic donor lymphocyte infusion after T cell-depleted allogeneic stem cell transplantation in acute leukemia patients
Source: Ann Hematol. 2023 Mar 7;102(5):1203–13. doi: 10.1007/s00277-023-05145-1 (PMC10102042; doi:10.1007/s00277-023-05145-1)
Supplement: Supplementary file 1 — Supplementary file1 (DOCX 18 kb) [file 277_2023_5145_MOESM1_ESM.docx]

**Supplement I: Transplantation details and the definitions used for high-risk leukemia**

For all patients in vitro TCD of the stem cell product was performed, either by incubation of the graft with alemtuzumab (20 mg) for 30 minutes at room temperature under continuous agitation, also known as ‘Campath in the bag’, or by CD34 cell selection. Patients up to the age of 60 years were transplanted with a myeloablative conditioning regimen, consisting of cyclophosphamide 60 mg/kg intravenously (i.v.) for 2 days, and TBI 9 Gy or busulphan 3.2 mg/kg i.v. for 4 days. In addition to this standard myeloablative conditioning, patients transplanted with a donor other than a 10/10 matched sibling donor received pretransplant alemtuzumab 15 mg i.v. for 2 days, and cyclosporine 3 mg/kg i.v. as GvHD prophylaxis starting at day -1. Cyclosporine was tapered from day 60 in the absence of GvHD.

For older or frail patients a RIC regimen was used, originally consisting of fludarabine 30 mg/m 2 i.v. or 50 mg/m 2 orally for 6 days, busulfan 3.2 mg/kg i.v. for 2 days, and horse-derived ATG (Lymphoglobulin) 10 mg/kg/day i.v. for 4 days. After Lymphoglobulin was withdrawn from the market in 2008, subsequent patients received alemtuzumab 15 mg i.v. for 2 days. From October 2009, patients with an unrelated donor and a RIC regimen additionally received rabbit-derived ATG (Thymoglobulin) 1-2 mg/kg/day i.v. for 1 day additionally. No additional

pharmacologic GvHD prophylaxis was given after RIC alloSCT.

The day of granulocyte engraftment was defined as the first of 3 consecutive days with absolute granulocyte counts >0.5x10 9 /L. Routine follow-up performed every 3 months during the first 2 years after alloSCT and additionally at 6 weeks after each DLI included bone marrow (BM) aspiration with morphological examination, immunophenotyping by flow cytometry, and molecular measurement of chimerism with a sensitivity of 1-2%. Upon clinical or laboratory signs of disease progression, additional BM examination and radiological assessment were performed.

High risk of early relapse with respect to our DLI strategy was defined according to applicable national Dutch recommendations for acute myeloid and lymphoblastic leukemia [20-23] . Specifically, high-risk ALL was defined by high leukocyte count at diagnosis (&gt;30x10 9 /L in B-ALL and &gt;100x10 9 /L in T-ALL), failure to achieve CR after first induction therapy, and/or unfavorable karyotypes (t(9;22), t(4;11), hypodiploidy, or complex abnormalities). High-risk AML was defined by therapy-related AML, presence of monosomal karyotype and/or abn3q26 (EVI1)), persistence of genetic abnormalities despite morphologic CR at time of alloSCT, and/or relapsed acute leukemia after previous curative induction chemotherapy. Leukemia patients not fulfilling these high-risk criteria served as the control group for this analysis.

**Supplement II:** Detailed descriptions of all study endpoints

Non-relapse mortality (NRM) was defined as death after alloSCT before any relapse. Relapse was defined as the reappearance of ≥5% blasts in BM by morphology, presence of >1% blasts in peripheral blood or the development of extra-medullary disease. Moderate to severe GvHD was defined as the presence of GvHD requiring sIS for more than 2 weeks, or GvHD related death. GvHD related death was defined as death due to (the treatment of) GvHD. Cause of death was adjudicated retrospectively by a panel of hematologists, based on standardized case reports.

Treatment success was defined as being alive without previous relapse post-alloSCT or current use of sIS.

The primary endpoint for the feasibility analysis was defined as the percentage of patients with high-risk leukemia for whom the first prophylactic DLI at 3 or 6 months after transplantation was requested and who actually received this DLI. Primary outcome for the toxicity analysis was the cumulative incidence of moderate to severe GvHD in the period between the first and second prophylactic DLI. To evaluate to what extent this toxicity was due to the low-dose DLI, the intervention group was compared to a control group. The intervention group consisted of all high-risk leukemia patients who received the planned early low-dose DLI at 3 (median 3.25, range 2.92-4.66) months after TCD-alloSCT. The control group consisted of patients being alive and without relapse or GvHD at 3.25 months who were not intended to receive the low-dose DLI because they lacked the criteria for high-risk acute leukemia. As the median time between the first and second DLI was 3.12 months for the intervention group, for this analysis the follow-up time for the patients who did not receive the second prophylactic DLI was stopped 3.12 months after the first DLI to keep the at-risk periods equal. For the control group, similar start and stop dates were taken to avoid bias in the comparison. The toxicity analysis started at 3.25 months after TCD-alloSCT and stopped at 6.37 months after TCD-alloSCT. Relapse, death without relapse and administration of other types of DLI were included as competing risks for GvHD occurrence. Subgroup analysis was performed on the type of conditioning regimen and the degree of matching between donor and patient. All 220 acute leukemia patients, stratified by type of leukemia and risk category, were included in the analysis of clinical outcomes. Probabilities of treatment success, RFS and overall survival (OS) and cumulative incidences of relapse, NRM, and GvHD were estimated at one, three and five years after TCD-alloSCT.

**Supplement III:** Statistical analysis

The probability of treatment success at 1, 3 and 5 years after alloSCT was calculated using a Markov multi-state model. All patients started in the state ‘no sIS’ at time of transplantation, including patients receiving prophylactic but not therapeutic sIS. Prophylactic sIS was tapered and stopped in all patients within the first 6.5 months after transplantation. Patient could move from the state ‘no sIS’ to one of the following states: ‘sIS’ at time of start of sIS for GvHD treatment without any prior relapse, ‘relapse’ at time of relapse, or ‘death without relapse’ at time of death without any prior relapse. From the state ‘sIS’, patients could return to the state ‘no sIS’ if they stopped all sIS, or to the states ‘relapse’ or ‘death without relapse’. From the state ‘relapse’, patients could only enter the state ‘death after relapse’. Both ‘death’ states were absorbing, meaning that patients could never leave these states. The percentage of patients being alive without relapse who needed treatment with sIS at 1,3 and 5 years after alloSCT was calculated as a crude percentage. Statistical software used was SPSS, PASW Statistics 20, release 20.0.0 (2011) and R 3.1.0 ( http://www.r-project.org/foundation ) using the packages ‘prodlim’, ‘survival’, ‘cmprsk’ and ‘mstate’.

**Table 1a Supplemental Material: Baseline characteristics and engraftment data of the complete AML cohort**

| **Variable** | **Categories** | **AML snon-high-risk (n=115)** | **AML high-risk (n=45)** |
| --- | --- | --- | --- |
| Sex | Male | 61 (53%) | 27 (60%) |
|  | Female | 54 (47%) | 18 (40%) |
|  |  |  |  |
| Age at alloSCT | Median age in years (range) | 52 (21-72) | 53 (19-71) |
|  | <45yr | 36 (31%) | 9 (20%) |
|  | 45-55yr | 32 (28%) | 16 (36%) |
|  | 55-65yr | 33 (29%) | 11 (24%) |
|  | >65yr | 14 (12%) | 9 (20%) |
|  |  |  |  |
| Donor relation | HLA-identical sibling | 48 (42%) | 15 (33%) |
|  | Unrelated | 67 (58%) | 30 (67%) |
|  |  |  |  |
| 10 out 10 matching | Yes | 100 (87%) | 40(89%) |
|  | No | 15 (13%) | 5 (11%) |
|  |  |  |  |
| Conditioning regimen | MA | 64 (56%) | 25 (56%) |
|  | RIC | 51 (44%) | 20 (44%) |
|  |  |  |  |
| Performance status at alloSCT | WHO score 0-1 | 95 (83%) | 41 (91%) |
|  | WHO score >1 | 10 (9%) | 2 (4%) |
|  | Missing | 10 (9%) | 2 (4%) |
|  |  |  |  |
| Engraftment neutrophils | Median days (range) | 16 (11-69) | 15 (9-29) |
|  |  |  |  |
| Rejection | Yes | 1 | 0 |
|  |  |  |  |
| GvHD prophylaxis | None | 78 (68%) | 31 (69%) |
|  | Cyclosporine A | 37(32%) | 14 (31%) |

*Abbreviations: alloSCT= allogeneic stem cell transplantation; MA=myeloablative; RIC=reduced intensity; GvHD=graft-versus-host-disease*

**Table 1b Supplemental Material: Baseline characteristics and engraftment data of the complete ALL cohort**

| **Variable** | **Categories** | **ALL snon-high-risk (n=22)** | **ALL high-risk (n=38)** |
| --- | --- | --- | --- |
| Sex | Male | 15 (68%) | 29 (76%) |
|  | Female | 7 (32%) | 9 (24%) |
|  |  |  |  |
| Age at alloSCT | Median age in years (range) | 29 (20-71) | 40 (18-65) |
|  | <45yr | 17 (77%) | 25 (66%) |
|  | 45-55yr | 1 (4.5%) | 7 (18%) |
|  | 55-65yr | 3 (14%) | 5 (13%) |
|  | >65yr | 1 (4.5%) | 1 (3%) |
|  |  |  |  |
| Donor relation | HLA-identical sibling | 10 (46%) | 12 (32%) |
|  | Unrelated | 12 (54%) | 26 (68%) |
|  |  |  |  |
| 10 out 10 matching | Yes | 15 (68%) | 30 (79%) |
|  | No | 7 (32%) | 8 (21%) |
|  |  |  |  |
| Conditioning regimen | MA | 19 (86%) | 33 (87%) |
|  | RIC | 3 (14%) | 5 (13%) |
|  |  |  |  |
| Performance status at alloSCT | WHO score 0-1 | 20 (91%) | 32 (84%) |
| (N=22/ N=35) | WHO score >1 | 2 (9%) | 3 (8%) |
|  | Missing | 0 | 3 (8%) |
|  |  |  |  |
| Engraftment neutrophils | Median days (range) | 17 (12-31) | 16 (10-48) |
|  |  |  |  |
| Rejection | Yes | 0 | 0 |
|  |  |  |  |
| GvHD prophylaxis | None | 12 (55%) | 15 (39%) |
|  | Cyclosporine A | 10 (45%) | 23 (61%) |

*Abbreviations: alloSCT= allogeneic stem cell transplantation; MA=myeloablative; RIC=reduced intensity; GvHD=graft-versus-host-disease*

**Table 2 Supplemental Material: Percentages of patients alive without relapse at 1, 3 and 5 years after alloSCT who still needed sIS at that time**

| **Group** | **1 year** | **3 year** | **5year** |
| --- | --- | --- | --- |
|  | % sIS (total pat) | % sIS (total pat) | % sIS (total pat) |
| Total cohort (N=220) | 16% (122) | 3% (107) | 1% (96) |
|  |  |  |  |
| AML non-high-risk (N=115) | 17% (70) | 5% (59) | 2% (53) |
| AML high-risk (N=45) | 14% (14) | 0% (13) | 0% (13) |
| ALL non-high-risk (N=22) | 21% (14) | 0% (13) | 0% (11) |
| ALL high-risk (N=38) | 8% (24) | 0% (22) | 0% (19) |

Abbreviations*: alloSCT= allogeneic stem cell transplantation; N=number of patients;% sIS= percentage of patients alive without relapse who still needed sIS; Total pat= total number of patients alive without relapse under observation*
